# Circadian Regulation of Lipid Metabolism during Pregnancy

**DOI:** 10.3390/ijms252111491

**Published:** 2024-10-26

**Authors:** Yujie Luo, Xinhang Meng, Liyuan Cui, Songcun Wang

**Affiliations:** Laboratory for Reproductive Immunology, Hospital of Obstetrics and Gynecology, Fudan University Shanghai Medical College, Shanghai 200011, China; 23211250013@m.fudan.edu.cn (Y.L.); 22211250012@m.fudan.edu.cn (X.M.)

**Keywords:** circadian rhythm, lipid metabolism, pregnancy, pregnancy-related diseases, sleep

## Abstract

A cluster of metabolic changes occur to provide energy for fetal growth and development during pregnancy. There is a burgeoning body of research highlighting the pivotal role of circadian rhythms in the pathogenesis of metabolic disorders and lipid homeostasis in mammals. Perturbations of the circadian system and lipid metabolism during gestation might be responsible for a variety of adverse reproductive outcomes comprising miscarriage, gestational diabetes mellitus, and preeclampsia. Growing studies have confirmed that resynchronizing circadian rhythms might alleviate metabolic disturbance. However, there is no clear evidence regarding the specific mechanisms by which the diurnal rhythm regulates lipid metabolism during pregnancy. In this review, we summarize previous knowledge on the strong interaction among the circadian clock, lipid metabolism, and pregnancy. Analyzing the circadian clock genes will improve our understanding of how circadian rhythms are implicated in complex lipid metabolic disorders during pregnancy. Exploring the potential of resynchronizing these circadian rhythms to disrupt abnormal lipid metabolism could also result in a breakthrough in reducing adverse pregnancy outcomes.

## 1. Introduction

Complicated maternal–fetal crosstalks contribute to a successful pregnancy, during which the uterus gradually transforms into a specialized niche, fulfilling the needs of a growing semi-allogeneic fetus and maintaining maternal–fetal tolerance [1,2]. An optimum supply of lipids, as one of the most essential nutrients in pregnancy, has important implications for the future health of gravidas and fetuses [3,4]. Intricate changes in lipid metabolism were observed in pregnant women, characterized by fat accumulation, maternal physiological hyperlipidemia, and increased tissue lipolysis [3,5]. Approximately 45% of reproductive-age women could be affected by lipid abnormalities [6]. It has been demonstrated that disturbances in maternal lipid profile could cause gestational diabetes mellitus (GDM), preeclampsia (PE), preterm birth, and fetal growth disorders [7,8]. Moreover, various adverse effects of dyslipidemia in reproductive-age women could continue beyond the termination of pregnancy [9,10]. Therefore, it is essential to explore the factors of susceptibility and the underlying mechanisms of lipid metabolism disorders, which may supply novel ideas for the therapy of metabolic disorders and adverse pregnancy outcomes.

A circadian rhythm, a term first noticed by Halberg in 1959 to explain an internal physiological regularity, represents an endogenous oscillation approximated to the Earth’s daily rotation period [11], and it exists in the brain and peripheral organs, such as the liver, skeletal muscle, esophagus, and reproductive organs [11,12,13]. Circadian rhythms have been demonstrated to exert a pivotal influence on implantation, decidualization, and pregnancy maintenance [14,15]. In humans and rodents, disruption of the circadian rhythm throughout pregnancy increased the risk of implantation failure, spontaneous abortion, preterm birth, and intrauterine growth restriction [16,17,18,19]. Research has indicated that the deficiency of circadian rhythm-related transcription factors in mice brain and muscle aryl hydrocarbon receptor nuclear translocator-like protein 1 (Bmal1), circadian locomotor output cycles protein kaput (Clock), and period 1 (Per1) resulted in detrimental outcomes including implantation failure, miscarriage, and infertility [20,21].

The circadian rhythm precisely regulates important physiological activities, such as metabolic rhythm, sleep/wake cycles, and endocrine rhythm. Peroxisome proliferator-activated receptors (PPARs), as key transcriptional regulators of lipid metabolism, are regulated by circadian rhythm in the liver, muscle, and white and brown adipose tissue, hereby linking the circadian rhythm to the lipid metabolism [22,23,24]. However, the mechanism by which circadian rhythm affects lipid metabolism during pregnancy remains largely exclusive.

Based on the evidence presented above, the first half of the article provides an overview of the functional aspects of the circadian machinery and discusses current research connections between circadian clock genes and lipoproteins. In the second half, we focus on whether the circadian rhythm clock regulates lipid metabolism during pregnancy and the impact of their abnormalities on pregnancy outcomes.

## 2. The Molecular Components of the Circadian Clock

Circadian rhythm in the wild, entrained to 24 h through changing ambient cues, especially light, enables the human body to switch its behavior and physiology to anticipate and respond to associated changes in the external environment [25,26]. The changes in behavior and physiology, such as sleep deprivation, disrupted eating-time, and shift work, could markedly contribute to the disturbance of circadian rhythm in organisms [27,28,29,30,31], leading to the development and progression of several diseases, including pregnancy-related disorders [32,33]. A meta-analysis illustrated that pregnant women who worked rotating shifts, fixed night shifts, or longer hours could experience a miscarriage, preterm delivery, or give birth to low birth weight infants [16]. It is necessary to comprehend the formation mechanism of the circadian clock system before discussing the circadian regulation of lipid metabolism during pregnancy.

In mammals, the central clock that generates the circadian rhythm is located in the suprachiasmatic nucleus of the anterior hypothalamus, the prime pacemaker of the organism [34,35]. Neurons within the suprachiasmatic nucleus can directly or indirectly receive the retinal input of an electric pulse, which is the code of light information from the environment [36]. These neurons then synchronize the circadian rhythm in peripheral organs, such as adipose tissue, kidney, spleen, lung, liver, and leukocytes, via neural and humoral pathways [37], although peripheral clocks can also be entrained independently [38]. The master clock has also been shown to serve as the core regulator for diurnal rhythmic behavior, such as feeding and sleep [39,40,41]. Disturbances of circadian rhythms in pregnant individuals could be linked to alterations in the immune system as well as the endocrine system, such as the diurnal cortisol rhythm [42,43].

At the molecular level, the circadian rhythm is operated by auto-regulatory transcriptional–translational loops composed of a suite of molecular pacemakers, including Bmal1 (also called Mop3 or Arntl), Clock, Per, cryptochrome (Cry), Rev-erbα (encoded by nuclear receptor subfamily 1 group D member 1), and retinoic acid receptor-related orphan receptor (Ror) [44].

**Figure 1:**
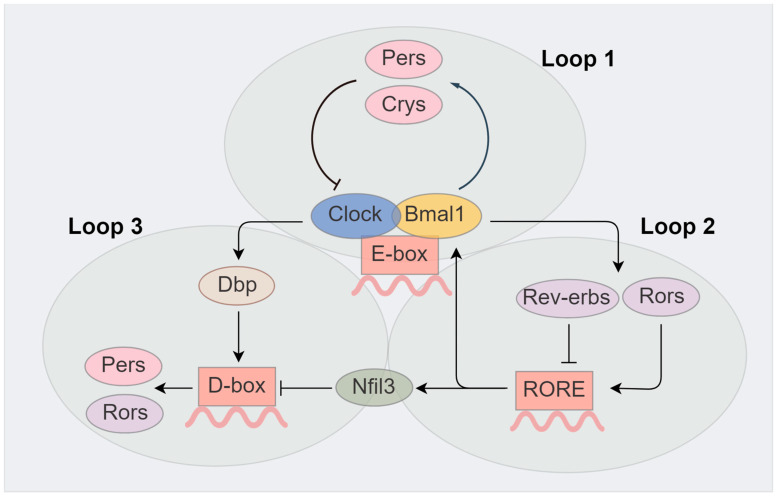
The key three loops in the core oscillator. There are three loops in the core oscillator, which constitute the basis for regulating the circadian rhythm of organisms. In the first loop, Bmal1/Clock heterodimers bind to E-box sites on the promoters of Pers and Crys to regulate their transcription and their heterodimers suppress the transcriptional regulatory function of Bmal1/Clock heterodimers by interacting with them. In the second loop, Bmal1/Clock heterodimers regulate the transcription of Rev-erbs and Rors, and their proteins regulate the transcription of Bmal1 by binding to RORE on the promoter region. In the third loop, Bmal1/Clock heterodimers regulate the Dbp transcription. Rev-erbs and Rors regulate the Nfil3 transcription. Dbp and Nfil3 regulate the transcription of Pers and Rors positively and negatively via binding D-box on the promoter region of it, respectively. Figures were created with Figdraw (www.figdraw.com, accessed on 7 July 2024).

The transcriptional–translational loops consist of three loops, which constitute the basis of circadian rhythm regulation of the organism (Figure 1).

In the first loop, the proteins of two core genes Bmal1 and Clock combine to form a heterodimer in the cytoplasm, transfer to the nucleus, and integrate with canonical enhancer-box (E-box) sites on the promoters of clock-controlled genes, such as Per, Cry, Rev-erbs, Rors, and albumin D-site-binding protein (Dbp), forming a positive side of the loop [44,45,46,47]. Per and Cry heterodimers, as a negative side of the loop, translocate into the nucleus to suppress the transcriptional regulatory function of Bmal1/Clock heterodimers by interacting with them [48,49], thereby interfering with the transcription of other circadian rhythm-related genes as well as their own [50,51]. The repressive effect of these factors lessens, owing to the reduced expression of central genes. Then, the transcriptional regulation of Bmal1/Clock heterodimers starts anew with another 24 h period [52].

In the second loop, Rors (Rorα, Rorβ, and Rorγ) and Rev-erbs (Rev-erbα and Rev-erbβ), activated by the Bmal1/Clock heterodimers, compete for Ror-binding elements (RORE) on the promoter region of Bmal1, thereby bringing about negative (Rev-erbs) and positive (Rors) regulatory impacts on the Bmal1 transcription [53,54,55]. In addition, this loop plays a pivotal role in circadian oscillators of multiple metabolic pathways including lipid metabolism [54]. Research has revealed that nuclear receptor Rev-erbα could drive the rhythmic expression of cholesterol-7alpha-hydroxylase, the rate-limiting enzyme in the pathway converting cholesterol to bile acids [56].

In the third loop, Bmal1/Clock heterodimers regulate the Dbp transcription [57] and Cry1 binding to E-box could delay the phase of Dbp expression [58]. Rev-erbs and Rors regulate the Nfil3 transcription. Dbp and Nfil3 regulate the transcription of Pers and Rors positively and negatively via binding to D-box on the promoter region, respectively [59,60]. Therefore, the organism accomplishes the diurnal rhythmic regulation based on these three loops.

## 3. Connections Between Circadian Clock Genes and Lipid Metabolism

The circadian rhythm was proposed to be highly relevant for metabolic disorders and could take part in maintaining lipid homeostasis in mammals. Growing studies confirmed that resynchronizing the circadian rhythm might alleviate metabolic disturbance. Here, we focus on several circadian clock genes that directly regulate lipid metabolism.

### 3.1. Bmal1

Bmal1 is a core constituent of the circadian clock in mammals and dysregulation of Bmal1 results in aberrant lipid metabolism. In hepatocyte-specific Bmal1 knockout mice, perivascular adipose tissue exhibited increased levels of triglyceridess (TGs) and free fatty acids (FFAs), as well as elevated mRNA expression of some crucial metabolic genes, including PPARγ, adiponectin, and lipoprotein lipase [61]. These are in line with a previous study that reported that hepatic Bmal1-deficient mice could result in declined transcript levels of PPAR signaling, which is a kind of metabolic pathway related to lipid metabolism [62]. Furthermore, both fatty acid oxidation (FAO) and de novo lipogenesis could be simultaneously suppressed in ethanol diet-fed mice with liver-specific deletion of Bmal1, which could be restored by promoting the PPARα pathway [63]. Bmal1 has been proposed to be an upstream regulator of PPARα gene expression, and the latter could interact with the peroxisome proliferator-activated receptor γ coactivator 1α, thereby facilitating the FAO [64,65,66]. It might be intriguing to explore the mechanisms by which PPARs connect circadian clock genes to lipid metabolism.

Interestingly, there are also other mediators by which the circadian gene Bmal1 plays roles in lipid metabolism. By interacting with the enhancer of the zeste homolog, Bmal1 could result in reduced expression of glycerol-3-phosphate acyltransferase mitochondrial, a crucial enzyme in lipid biosynthesis, thereby leading to decreased triacylglycerol and lysophosphatidic acid (LPA) [67]. It has been confirmed that Bmal1 regulates microsomal triglyceride transfer protein and ATP binding cassette family G protein 5 or protein 8 through the small heterodimer partner and Gata4, respectively, thereby affecting lipoprotein production and biliary cholesterol excretion [68]. Furthermore, hepatic-specific Bmal1 inactivation contributed to increased plasma low-density lipoprotein/very-low-density lipoprotein cholesterol (LDL-C/VLDL-C) levels as a result of the dysfunction in proprotein convertase subtilisin/kexin type 9 [69]. It has also been reported that the oxidation capacity of fatty acids increased following the deletion of Bmal1 in both untreated and chronic insulin treatment cells, suggesting that Bmal1 reduced the utilization of fatty acids [70].

### 3.2. Clock

Clock is another critical component of the circadian rhythm in modulating lipid metabolism, and disruption of Clock could lead to lipid metabolic disorders. Clock mutant mice fed with cholesterol/cholic acids could accumulate a higher amount of cholesterol in the liver due to the reduced expression of cholesterol-7alpha-hydroxylase [56]. Compared with wild-type mice, Clock^Δ19/Δ19^ double mutant mice have been found to augment the absorption of intestinal cholesterol, increase the uptake of macrophage-modified lipoproteins, and attenuate the efflux of cholesterol from macrophages, which eventually lead to atherosclerosis [71]. Moreover, it was reported that homozygous Clock mutant mice showed greatly declined circadian feeding rhythms and a metabolic syndrome of hyperglycemia, hyperlipidemia, and hyperleptinemia [72]. A mechanistic link has been found between the perturbation of the Clock gene and the progression of nonalcoholic fatty liver disease [73]. CLOCK knockout mice presented disruptions of proliferation and adipogenesis markers within white adipose tissue, while Clock^Δ19/Δ19^ double mutant mice exhibited elevated fatty acid uptake attributed to heightened hypoxia-inducible factor 1α protein levels [56,74]. Additionally, the genetic perturbation of molecular clocks in Clock-deficient mice could result in the elimination of diurnal oscillations of triglycerides [75]. Persistent hypertriglyceridemia, increased lipid absorption, and a higher level of microsomal triglyceride transfer protein expression were also reported in Clock mutant mice in comparison to wild-type mice [76,77].

### 3.3. Rev-Erbs

The nuclear receptor Rev-erbα and its close homolog Rev-erbβ are transcriptional repressors in the circadian rhythm of mammals. Abnormal expression of Rev-erbs could result in dysregulation of lipid and energy metabolism. Rev-erbα/β-agonist-treated mice exhibited a decrease in fat mass, a 12% decline in plasma TG, and a 47% reduction in plasma total cholesterol [78]. It has been shown that Rev-erbα took part in the circadian modulation of sterol regulatory element-binding protein activity via the circadian transcription of insulin-induced gene 2, thereby involving the regulation of cholesterol and lipid metabolism [79,80]. The Rev-erbα protein recruits the histone de-acetylases 3 to take part in lipid metabolism, and dysfunction of either could cause hepatic steatosis [81,82]. In addition, Rev-erbs effectively inhibited the expression of lipogenic enzymes, such as stearoyl-CoA desaturase 1 and fatty acid synthase, thereby influencing the synthesis of fatty acid [78]. It has also been reported that Rev-erbs could play a vital role in the regulation of lipid metabolism via interacting with other circadian clock genes. For example, the Bmal1–Rev-erb–choline kinase axis could regulate hepatic phosphatidylcholine, suggesting that an intact circadian system is of particular importance for phospholipid metabolism [83].

### 3.4. Per

The transcriptional factors Per1 and Per2 play important roles in a series of physiological processes, including the circadian regulation of lipid metabolism. Studies have proposed that the circadian cycle of phospholipid biosynthesis in cultured fibroblasts disappeared after the deletion of Per1 [84,85]. Deletion of Per1 decreased TG accumulation in the serum and liver, while overexpression of Per1 up-regulated the expression of lipogenesis-related genes, including PPARα and its target genes [86].

Lipidomic profiling also showed that the clock gene Per2 is essential for normal lipid metabolism in white adipocyte tissue [87]. It has been confirmed that saturated and mono-unsaturated very-long-chain fatty acids levels augmented, while TG and white adipose tissue decreased in Per2 knockout mice compared with those in controls [87]. Moreover, Per2 could interact directly with PPARα, modify its activity via competing with the activities of PPARγ, and alter lipid metabolism [87].

### 3.5. Other Circadian Clock Genes

In addition, other clock genes might be involved in the regulation of lipid metabolism, such as Dec, Rors, and Nfil3. Dec1 and Dec2, the fifth clock gene family to regulate metabolic functions genes, inhibit lipogenesis in the liver via binding to the promoter region of sterol regulatory element-binding protein 1c, thereby modulating fatty acid synthesis [88,89]. Ror family members, required for cell-autonomous clock function in the liver, could regulate the expression of circadian rhythm clock genes via interacting with the peroxisome proliferator-activated receptor γ coactivator 1α [90]. Nfil3, an important factor of the circadian clock system, acts as a positive regulator of adipogenesis. Upregulated Nfil3 in the aging thymus was associated with steroid and adipogenesis process of thymic epithelial cells via promoting epithelial–mesenchymal transition [91]. Moreover, Nfil3, as a key pro-adipogenic transcription factor, could trans-repress cyclooxygenase-2 in glucocorticoid-associated adipocyte differentiation [92].

Taken together, these results emerged that circadian clock proteins controlled lipid metabolic homeostasis, possibly through specific transcription factors containing PPARs, sterol regulatory element-binding protein, and peroxisome proliferator-activated receptor γ coactivator 1α.

## 4. Circadian Regulation of Lipid Metabolism During Pregnancy

A disturbed clock during pregnancy has been identified as a contributing factor in disorders of carbohydrate metabolism as well as abnormal glucose metabolism [93]. There is also a cluster of evidence that disruption of the circadian clock may be associated with lipid metabolism. It has been proposed that the dysfunction of the circadian rhythm leads to changes in circulating energy consumption and lipid metabolism, thereby promoting the occurrence of liver cancer [94]. However, the exact molecular mechanisms underlying this effect during pregnancy have yet to be elucidated. Herein, we provide a comprehensive analysis of the physiological variation in the circadian rhythm and characteristics of lipid profile during pregnancy. Furthermore, we also concentrate on the interaction between disrupted lipid metabolism, dysregulated circadian rhythm, and several adverse pregnancy results.

### 4.1. Physiological Changes in Circadian Rhythm During Pregnancy

**Figure 2:**
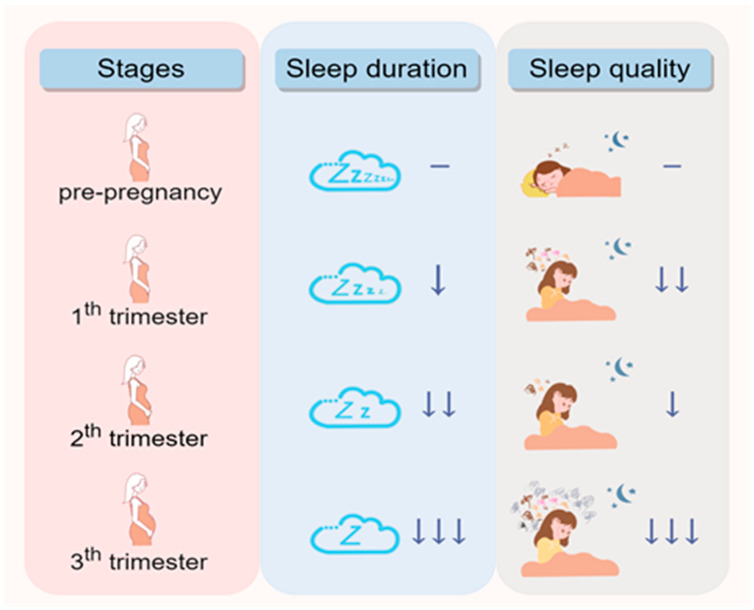
Changes in sleep patterns during pregnancy. Compared to pre-pregnancy, significant changes in sleep duration and sleep quality were observed during pregnancy. ‘↓’ means shorter sleep duration or poorer sleep quality compared with pre-pregnancy. ‘—’ means normal sleep duration and quality in pre-pregnancy. Figures were created with Figdraw.

Sleep pattern in reproductive-age women is frequently affected by dramatic changes in hormone secretion, cardiovascular function, fetal movement, and frequent awakenings to urinate caused by a growing uterus [95]. Thus, significant changes in sleep pattern were observed during pregnancy (Figure 2). A midline estimating statistic of rhythm in the middle and late trimester is higher than that in the early trimester, reaching a peak at approximately 32 weeks of gestation [96]. Changes in sleep pattern during pregnancy have a similar result with the midline estimating statistic of rhythm [97]. The feature of sleep pattern in the first trimester of gestation involves shorter sleep latency, and poorer sleep quality compared with those in pre-pregnancy [98,99]. Temporarily improved sleep quality was observed in the mid-trimester gestation [99], while sleep quality could worsen from the 2nd to the 3rd trimester [100]. Moreover, sleep quality during the third trimester is even worse than that in first trimester [99,101]. Sleep duration decreased from the first to second trimester [102], with a shorter sleep duration in the third trimester than in the first [101]. It has been reported that a shorter sleep duration was associated with advancing gestational age [99,103]. However, recent research differing from previous reports indicated that sleep duration was prolonged in all trimesters compared to pre-pregnancy, which could be attributed to the baseline measurement [99]. To date, as there are still no clear mechanisms by which pregnancy exerts impacts on sleep pattern, it is fascinating to explore mutual regulation between circadian clock proteins and sleep patterns during pregnancy. Such data on sleep changes in healthy gestation might also be used as a baseline to identify sleep-related risk factors for adverse pregnancy outcomes.

Research has demonstrated that the total energy intake of the 3rd trimester increased by 9% (185 kcal/day) compared to the 1st trimester [104]. An increased energy supply is necessary for pregnancy to support various activities, particularly in relation to lactation, placental and fetal development, and maternal physiological adaptations [105]. However, late sleep midpoint (after 5:00 am) and short sleep duration during the second-trimester gestation correlate with gestational diabetes [106,107]. Similarly, other reports showed that a short sleep duration during pregnancy reduced the risk of glucose tolerance and increased the risk of hyperglycemia [108,109]. Long sleep durations were also implicated in placental abruption, while a short sleep duration has been linked to increased risks of placental abruption and preterm birth [110,111]. However, the mechanisms of energy metabolism abnormalities caused by sleep pattern changes during pregnancy are still unclear.

### 4.2. Characteristics of Lipid Metabolism During Pregnancy

Pregnancy is a unique physiological period in a woman’s life, during which a cluster of metabolic changes occur to provide energy for fetal growth and development. Two states, anabolic and catabolic, could be observed in maternal lipid metabolism during pregnancy [5,112,113]. The anabolic state occurs during the early and middle trimesters, as the body prepares for the fetal energy needed in the later stage of pregnancy. A decline in fat accumulation appears during the last trimester, resulting in a transition from the anabolic phase into the catabolic phase [113]. During this period, the activity of lipoprotein lipase in the adipose tissue could surge or stabilize [114,115]. It could increase hydrolysis of circulating triacylglycerol and the production of lipid products such as 2-monoacylglycerol, non-esterified fatty acids, and glycerol, which are then taken up and used for TG re-synthesis [116].

Owing to the processes mentioned above, changes in lipid levels are presented in a trimester-dependent manner during pregnancy. In the early trimester of pregnancy, LDL-C, apolipoprotein B, total cholesterol, and TG levels decline moderately and increase from the second trimester until the end of term [117]. The TG level increases by approximately 50–100%, while total cholesterol and LDL-C levels rise by around 30–50% [118]. High-density lipoprotein cholesterol levels and apolipoprotein A1 augment by 20–40% from the early trimester onwards and reach a plateau at around 20–24 weeks [117]. Moreover, the maternal pancreatic beta-cell mass rises to enhance the secretion of insulin, which is considered as an important regulator of lipoprotein lipase and catalyzes the hydrolysis and uptake of circulating TG [119,120]. The progressive increase in insulin resistance during pregnancy is significantly driven by prolactin, estrogen, and progesterone [121]. Moreover, elevated maternal adiposity, particularly in the 1st trimester, could exacerbate insulin resistance and increase lipolysis by the 3rd trimester, and contribute to raised FFA concentrations [122]. In turn, the resultant increase in maternal FFA levels exacerbates insulin resistance via hindering glucose uptake and augmenting hepatic gluconeogenesis [121]. It is for this reason that de novo lipogenesis is enhanced during gestation, leading to lipid storage [123]. Recent research indicated that the pronounced unsaturation of phospholipids remained a stable attribute during the embryonic transition to the blastocyst stage, which could be indispensable for membrane structure and cell signaling [124]. Indeed, multiple other factors have impacts on maternal lipid levels and the magnitude of this variation in pregnancy, such as pre-pregnancy body mass index, diet, age, and race [125,126,127,128,129].

Undesirable pregnancy outcomes could occur when lipid levels in pregnancy go beyond the physiologic range and develop dyslipidemia [130,131,132]. A study with 5690 women has confirmed a positive association between maternal blood pressure and certain lipids, including TG, LDL-C, non-high-density lipoprotein cholesterol, and especially TG [133]. Moreover, the research revealed that lipid levels during the early trimester of pregnancy were strongly linked to an increased risk of cardiovascular burden for the mother, including preeclampsia (PE) and sustained hypertension [133]. In a retrospective study of 12,715 women, a strong connection between increased first-trimester TG and GDM and PE was also observed [134]. TG-rich lipoproteins and their remnants during pregnancy might be associated with arterial stiffness, endothelial dysfunction and inflammation, which ultimately lead to consequent placental vessel dysfunction [135]. Recently, a retrospective study of 881 women with a twin pregnancy showed a similar conclusion that TG in the early trimester of pregnancy was highly relevant for PE and GDM [136]. Furthermore, recent retrospective cohort research also demonstrated that pregnant women with hypertensive disorders in pregnancy (HDP) might have an increase in serum lipids during pregnancy, with the exception of a decrease in FFA levels [137]. It is crucial for researchers to continue studying the mechanisms behind these associations in order to develop effective interventions and treatments for pregnant women with dyslipidemia.

### 4.3. Circadian Dysregulation of Lipid Metabolism and Adverse Pregnancy Outcomes

**Figure 3:**
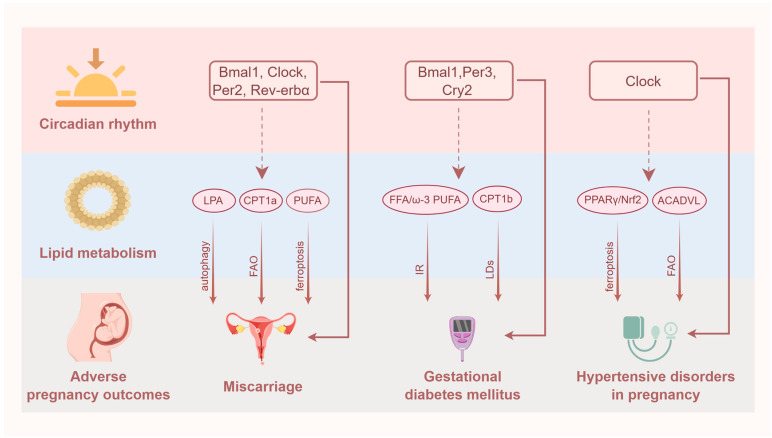
Circadian dysregulation of lipid metabolism and adverse pregnancy outcomes. Both circadian rhythm disorders and dysregulated lipid metabolism were proposed to result in adverse pregnancy outcomes. In miscarriage, circadian clock genes (Bmal1, Clock, Per2, and Rev-erbα) dysregulation could result in abortion and it has also been proven that LPA, CPT1a, and PUFA could influence pregnancy outcomes. Moreover, studies found that Bmal1 could affect the presence of LPA and FAO and Per2 could affect CPT1 expression, suggesting that miscarriages might be the result of lipid disorders caused by circadian clock perturbations. In GDM, lower transcript levels of Bmal1, Per3, and Cry2 were observed. FFA, ω3-PUFA, and CPT1b could lead to GDM via insulin resistance (IR) and the accumulation of lipid droplets (LDs). Meanwhile, Bmal1 depletion was shown to increase the FFA level. Thus, it is reasonable to speculate that circadian rhythm might participate in the development of GDM via regulating lipid metabolism. In HDP, the circadian gene Clock was reported to participate in the pathogenesis of PE. PPARγ/Nrf2 signaling pathway and acyl-CoA dehydrogenase very long chain (ACADVL) were also linked to the development of PE. Additionally, Clock mutant mice could increase lipid absorption and persistent hypertriglyceridemia, suggesting that HDP could be the result of lipid disorders caused by circadian disruption. Further studies should investigate whether adverse pregnancy outcomes are caused by circadian dysregulation of lipid metabolism and should elucidate the potential mechanisms. Solid line: confirmed; dashed line: partial confirmation. Figures were created with Figdraw.

Multiple organs of the human body are involved in lipid metabolism, and these organs are controlled by the circadian clock to regulate the body’s metabolic functions. Previous studies have shown the interlocking of dyslipidemia and circadian disorders with pregnancy-related diseases, respectively, which might contribute to the treatment of pregnancy complications. In the next section, we will discuss the link between circadian rhythm disorders and dysregulated lipid metabolism in several adverse pregnancy outcomes, including miscarriage, GDM, and HDP (Figure 3).

#### 4.3.1. Miscarriage

Miscarriage, a term for the loss of an intrauterine pregnancy up to 20–24 weeks of gestation, is the most common complication of pregnancy [138]. A remarkable association has been illustrated between lipid metabolism and miscarriage. For example, a defective LPA–autophagy axis could increase miscarriage risk by restricting decidual macrophage residence. LPA could alleviate embryo resorption in the spontaneous abortion model [139,140]. Meanwhile, a reduction in carnitine palmitoyltransferase 1A (CPT1a), a rate-limiting enzyme in the FAO pathway, has been shown to correlate with insufficient decidualization, heightening the likelihood of miscarriage [141]. Studies also suggested that the integration of polyunsaturated fatty acids (PUFAs) into membrane phospholipids promoted the creation of peroxides, thereby inducing ferroptosis [142,143,144]. Of note, decidual stromal cell ferroptosis could be implicated in the pathogenesis of recurrent pregnancy loss (RPL) [145].

In addition, research demonstrated that both decidualization and implantation could be affected by circadian genes [14,20,21]. Bmal1 could activate the migration and invasion of trophoblasts in RPL through the specificity protein 1–DNA 5′-cytosine-methyltransferases 1/DAB2 interaction protein axis, revealing a link between Bmal1 and RPL [146]. Apoptosis in mouse embryonic stem cells could be induced via downregulation of Clock, leading to an increase in miscarriage [147]. Per2 dysfunction does not synchronize aperiodic decidual gene expression with the initiation of endometrial proliferation, thereby resulting in RPL [148]. A declined implantation number, deficient decidualization, and increased abortion rate were observed in the sleep disorder models compared with those in normal mice, as decidualization could be regulated by the circadian gene Rev-erbα via the interleukin 6–progesterone receptors–CCAAT/enhancer-binding protein β pathway [149]. Moreover, we previously found that melatonin could reverse inflammation and decidualization resistance, indicating that the melatonin–melatonin receptor 1 signal might take part in pregnancy via regulating decidualization [150]. Numerous studies have provided evidence of the connection between sleep disorders and RPL, but the underlying mechanisms remain largely unclear.

Notably, while both the disturbance of Bmal1 and deficiency of LPA affected miscarriage, a link between the circadian gene Bmal1 and the lipid mediator LPA has been found [67], suggesting that miscarriage might be the result of lipid disorders caused by circadian clock perturbations. Per2 knockout in mice could affect lipid metabolism through altering the expression of CPT1 [151], while CPT1 has been shown to correlate with insufficient decidualization, heightening the likelihood of miscarriage and lipid disorders caused by circadian disruption. As a strong association has been observed among the circadian clock, lipid metabolism, and miscarriage, future studies should explore whether the miscarriage resulted from abnormal lipid metabolism induced by circadian disruption.

#### 4.3.2. GDM

GDM, one of the most prevalent metabolic disorders in gestation, denotes the development of glucose intolerance during pregnancy [152]. The prevalence of GDM in China was reported to be 19.7%, and has shown an upward trend during the past few years [153]. It has been proposed that lipid metabolism is also an indispensable factor for the development of GDM, except for glycometabolism. In GDM women, proper supplementation of omega-3 PUFA could decrease insulin resistance and inflammation, thereby decreasing the risk of undesirable reproductive outcomes [154,155]. Both acute and chronic increases in FFA could induce insulin resistance, as a study has shown that β-cell compensatory responses might be impeded along with the chronic increase in FFA levels [32,156]. FFA levels in pregnant women with GDM varied dramatically during gestational periods, with the majority increasing from early to mid-pregnancy and gradually declining in late pregnancy [32]. In addition, studies have confirmed that α-β hydrolase domain-containing protein 5/CPT1b could regulate the accumulation of lipid droplets in trophoblast cells, which is the feature of GDM [157].

Likewise, a growing body of research has proven the notion that impairment of the diurnal system, particularly concerning sleep disorders, plays a pathogenic role in GDM patients. A meta-analysis of seven studies revealed that extreme sleep durations (long or short sleep) in the early and mid-trimester could lead to a 1.35-fold increase in the risk of GDM [158]. Another meta-analysis of 16 studies, including 2,551,017 pregnant women and 142,103 GDM cases, to a great extent arrived at a similar result that an increased risk of GDM could be related to short or long sleep durations [159]. In addition, poor sleep quality, snoring, and even obstructive sleep apnea syndrome could be related to an increased prevalence rate of GDM [159]. Compared with controls, lower transcript levels of Bmal1, Per3, and Cry2 were observed in GDM women, indicating that the deranged expression of the circadian clock-related genes are strongly linked to the occurrence of GDM [160].

Additionally, Bmal1 depletion enhanced FFA levels, including saturated and mono-unsaturated very-long-chain fatty acids [61]. Sleep fragmentation or intermittent hypoxia could increase the risk of GDM via declining insulin sensitivity or elevating plasma glucose levels [161,162]. Thus, it is reasonable to speculate that circadian rhythm might participate in the development of GDM via regulating lipid metabolism as multiple studies have reported that both lipid metabolism and the circadian clock could affect GDM.

#### 4.3.3. HDP

HDP is categorized into four subtypes based on disease severity: gestational hypertension, chronic hypertension, PE, and chronic hypertension with superimposed preeclampsia. It was revealed that 15.3% of pregnant women could be affected by HDP, with PE being the most common subtype accounting for 54.9% of cases [33,163]. In recent years, several studies have provided accumulating evidence about the impact of lipid metabolism on the risk of HDP. For instance, perfluoroalkyl substances interfered with trophoblast invasion, inflammation, and lipid homeostasis by interacting with PPARs, thereby raising the incidence of PE [164]. Previous research has illustrated that PPARγ/Nrf2 signaling impacted ferroptosis by regulating lipid oxidation, which alleviated PE development [165]. The increased expression of acyl-CoA dehydrogenase very long chain involved in the FAO pathway could contribute to the development of PE through gluconeogenesis [166]. In women with PE, lower expression of genes involved in placental FAO and transport was exhibited compared with healthy women [167]. On the contrary, early and late-onset PE were also thought to profoundly affect maternal lipid metabolism, potentially contributing to increased cardiovascular risk later in life [168]. Thus, figuring out the relationship between lipid metabolism and the development of HDP is possibly needed in future research.

Meanwhile, the impact of the circadian system on the HDP occurrence has also been emphasized. Recent cohort research showed that circadian rhythm disturbance caused by skipping breakfast during gestation was related to the development of HDP [169]. It has been shown that the circadian gene Clock modulated the biological behavior of trophoblasts via hypoxia, participating in the pathogenesis of PE [170]. While extreme sleep duration and poor sleep quality in pregnancy augmented the risk of PE, sleep disorders among pregnant women comprising restless leg syndrome, subjective sleep-disordered breathing, and sleep apnea syndrome were also markedly associated with gestational hypertension and PE [171]. In addition, shift work during pregnancy was exhibited to increase the risk of PE [172,173]. Such major studies suggested that overall sleep disturbances in the gestation increased the risk of HDP occurrence, though the mechanisms underlying were poorly explained.

Significantly, it was observed that Clock mutant mice caused increased lipid absorption and persistent hypertriglyceridemia [72]. Future studies should investigate whether the circadian rhythm might modulate HDP via the lipid metabolism pathway.

## 5. Conclusions

Insights into the circadian clock and lipid metabolism have made significant advances during the past several decades. Dysregulation of the circadian clock could affect lipid synthesis and FAO, while dysfunction of this process could cause adverse pregnancy outcomes. Indeed, an increasing number of circadian rhythm studies have provided important insights that inspired us to correlate the expression of the circadian clock gene with lipid metabolism during pregnancy within the holistic understanding of the involved molecular mechanisms. However, we are only beginning to understand the systems and mechanisms, and clearly require additional experimentation to further broaden our comprehension of circadian rhythm and lipid metabolism during pregnancy. Hence, new insights that target key circadian clock genes with the intention of treating adverse pregnancy outcomes may provide more effective strategies, pharmacological approaches, and improved guidance for optimizing pregnancy development.

## References

[1] Vento-Tormo R., Efremova M., Botting R.A., Turco M.Y., Vento-Tormo M., Meyer K.B., Park J.-E., Stephenson E., Polański K., Goncalves A. (2018). Single-Cell Reconstruction of the Early Maternal–Fetal Interface in Humans. Nature.

[2] Menkhorst E., Winship A., Sinderen M.V., Dimitriadis E. (2016). Human Extravillous Trophoblast Invasion: Intrinsic and Extrinsic Regulation. Reprod. Fertil. Dev..

[3] Emet T., Ustüner I., Güven S.G., Balık G., Ural U.M., Tekin Y.B., Sentürk S., Sahin F.K., Avşar A.F. (2013). Plasma Lipids and Lipoproteins during Pregnancy and Related Pregnancy Outcomes. Arch. Gynecol. Obstet..

[4] Chavan-Gautam P., Rani A., Freeman D.J. (2018). Distribution of Fatty Acids and Lipids During Pregnancy. Adv. Clin. Chem..

[5] Herrera E. (2002). Lipid Metabolism in Pregnancy and Its Consequences in the Fetus and Newborn. Endocrine.

[6] Pugh S.J., Schisterman E.F., Browne R.W., Lynch A.M., Mumford S.L., Perkins N.J., Silver R., Sjaarda L., Stanford J.B., Wactawski-Wende J. (2017). Preconception Maternal Lipoprotein Levels in Relation to Fecundability. Hum. Reprod..

[7] Mossayebi E., Arab Z., Rahmaniyan M., Almassinokiani F., Kabir A. (2014). Prediction of Neonates’ Macrosomia with Maternal Lipid Profile of Healthy Mothers. Pediatr. Neonatol..

[8] Spracklen C.N., Smith C.J., Saftlas A.F., Robinson J.G., Ryckman K.K. (2014). Maternal Hyperlipidemia and the Risk of Preeclampsia: A Meta-Analysis. Am. J. Epidemiol..

[9] Metwally M., Li T.C., Ledger W.L. (2007). The Impact of Obesity on Female Reproductive Function. Obes. Rev..

[10] Jungheim E.S., Travieso J.L., Hopeman M.M. (2013). Weighing the Impact of Obesity on Female Reproductive Function and Fertility. Nutr. Rev..

[11] Persson P.B., Bondke Persson A. (2019). Circadian Rhythms. Acta Physiol..

[12] Harfmann B.D., Schroder E.A., Esser K.A. (2015). Circadian Rhythms, the Molecular Clock, and Skeletal Muscle. J. Biol. Rhythm..

[13] Hashimoto A., Uemura R., Sawada A., Nadatani Y., Otani K., Hosomi S., Nagami Y., Tanaka F., Kamata N., Taira K. (2019). Changes in Clock Genes Expression in Esophagus in Rat Reflux Esophagitis. Dig. Dis. Sci..

[14] Sen A., Sellix M.T. (2016). The Circadian Timing System and Environmental Circadian Disruption: From Follicles to Fertility. Endocrinology.

[15] Hirata M., He P.-J., Shibuya N., Uchikawa M., Yamauchi N., Hashimoto S., Hattori M. (2009). Progesterone, but Not Estradiol, Synchronizes Circadian Oscillator in the Uterus Endometrial Stromal Cells. Mol. Cell. Biochem..

[16] Cai C., Vandermeer B., Khurana R., Nerenberg K., Featherstone R., Sebastianski M., Davenport M.H. (2019). The Impact of Occupational Shift Work and Working Hours during Pregnancy on Health Outcomes: A Systematic Review and Meta-Analysis. Am. J. Obstet. Gynecol..

[17] Loy S.L., Cheung Y.B., Cai S., Colega M.T., Godfrey K.M., Chong Y.-S., Shek L.P.-C., Tan K.H., Chong M.F.-F., Yap F. (2020). Maternal Night-Time Eating and Sleep Duration in Relation to Length of Gestation and Preterm Birth. Clin. Nutr..

[18] Seron-Ferre M. (2019). Shift Work and Pregnancy: Night Light, Baby Not Right. J. Physiol..

[19] Varcoe T.J., Voultsios A., Gatford K.L., Kennaway D.J. (2016). The Impact of Prenatal Circadian Rhythm Disruption on Pregnancy Outcomes and Long-Term Metabolic Health of Mice Progeny. Chronobiol. Int..

[20] Liu Y., Johnson B.P., Shen A.L., Wallisser J.A., Krentz K.J., Moran S.M., Sullivan R., Glover E., Parlow A.F., Drinkwater N.R. (2014). Loss of BMAL1 in Ovarian Steroidogenic Cells Results in Implantation Failure in Female Mice. Proc. Natl. Acad. Sci. USA.

[21] Boden M.J., Kennaway D.J. (2006). Circadian Rhythms and Reproduction. Reproduction.

[22] Panda S., Antoch M.P., Miller B.H., Su A.I., Schook A.B., Straume M., Schultz P.G., Kay S.A., Takahashi J.S., Hogenesch J.B. (2002). Coordinated Transcription of Key Pathways in the Mouse by the Circadian Clock. Cell.

[23] Yang X., Downes M., Yu R.T., Bookout A.L., He W., Straume M., Mangelsdorf D.J., Evans R.M. (2006). Nuclear Receptor Expression Links the Circadian Clock to Metabolism. Cell.

[24] Adamovich Y., Aviram R., Asher G. (2015). The Emerging Roles of Lipids in Circadian Control. Biochim. Biophys. Acta.

[25] Bhadra U., Thakkar N., Das P., Pal Bhadra M. (2017). Evolution of Circadian Rhythms: From Bacteria to Human. Sleep Med..

[26] Khan S., Nabi G., Yao L., Siddique R., Sajjad W., Kumar S., Duan P., Hou H. (2018). Health Risks Associated with Genetic Alterations in Internal Clock System by External Factors. Int. J. Biol. Sci..

[27] West A.C., Bechtold D.A. (2015). The Cost of Circadian Desynchrony: Evidence, Insights and Open Questions. Bioessays.

[28] Harder L., Oster H. (2020). The Tissue Clock Network: Driver and Gatekeeper of Circadian Physiology: Circadian Rhythms Are Integrated Outputs of Central and Peripheral Tissue Clocks Interacting in a Complex Manner—From Drivers to Gatekeepers. Bioessays.

[29] Rijo-Ferreira F., Takahashi J.S. (2019). Genomics of Circadian Rhythms in Health and Disease. Genome Med..

[30] Wang F., Xie N., Wu Y., Zhang Q., Zhu Y., Dai M., Zhou J., Pan J., Tang M., Cheng Q. (2021). Association between Circadian Rhythm Disruption and Polycystic Ovary Syndrome. Fertil. Steril..

[31] Yaw A.M., Duong T.V., Nguyen D., Hoffmann H.M. (2021). Circadian Rhythms in the Mouse Reproductive Axis during the Estrous Cycle and Pregnancy. J. Neurosci. Res..

[32] Du H., Li D., Molive L.M., Wu N. (2024). Advances in Free Fatty Acid Profiles in Gestational Diabetes Mellitus. J. Transl. Med..

[33] Subki A.H., Algethami M.R., Baabdullah W.M., Alnefaie M.N., Alzanbagi M.A., Alsolami R.M., Abduljabbar H.S. (2018). Prevalence, Risk Factors, and Fetal and Maternal Outcomes of Hypertensive Disorders of Pregnancy: A Retrospective Study in Western Saudi Arabia. Oman Med. J..

[34] Blacher E., Tsai C., Litichevskiy L., Shipony Z., Iweka C.A., Schneider K.M., Chuluun B., Heller H.C., Menon V., Thaiss C.A. (2022). Aging Disrupts Circadian Gene Regulation and Function in Macrophages. Nat. Immunol..

[35] Ralph M.R., Foster R.G., Davis F.C., Menaker M. (1990). Transplanted Suprachiasmatic Nucleus Determines Circadian Period. Science.

[36] Golombek D.A., Rosenstein R.E. (2010). Physiology of Circadian Entrainment. Physiol. Rev..

[37] Kizaki T., Sato S., Shirato K., Sakurai T., Ogasawara J., Izawa T., Ohira Y., Suzuki K., Ohno H. (2015). Effect of Circadian Rhythm on Clinical and Pathophysiological Conditions and Inflammation. Crit. Rev. Immunol..

[38] Albrecht U. (2012). Timing to Perfection: The Biology of Central and Peripheral Circadian Clocks. Neuron.

[39] Top D., Young M.W. (2018). Coordination between Differentially Regulated Circadian Clocks Generates Rhythmic Behavior. Cold Spring Harb. Perspect. Biol..

[40] Allada R., Chung B.Y. (2010). Circadian Organization of Behavior and Physiology in Drosophila. Annu. Rev. Physiol..

[41] Erion R., King A.N., Wu G., Hogenesch J.B., Sehgal A. (2016). Neural Clocks and Neuropeptide F/Y Regulate Circadian Gene Expression in a Peripheral Metabolic Tissue. Elife.

[42] Guan Y., Xu M., Zhang Z., Liu C., Zhou J., Lin F., Fang J., Zhang Y., Yue Q., Zhen X. (2023). Maternal Circadian Disruption before Pregnancy Impairs the Ovarian Function of Female Offspring in Mice. Sci. Total Environ..

[43] Bedrosian T.A., Fonken L.K., Nelson R.J. (2016). Endocrine Effects of Circadian Disruption. Annu. Rev. Physiol..

[44] Xiang K., Xu Z., Hu Y.-Q., He Y.-S., Wu G.-C., Li T.-Y., Wang X.-R., Ding L.-H., Zhang Q., Tao S.-S. (2021). Circadian Clock Genes as Promising Therapeutic Targets for Autoimmune Diseases. Autoimmun. Rev..

[45] Schibler U., Sassone-Corsi P. (2002). A Web of Circadian Pacemakers. Cell.

[46] Reppert S.M., Weaver D.R. (2002). Coordination of Circadian Timing in Mammals. Nature.

[47] Amir M., Chaudhari S., Wang R., Campbell S., Mosure S.A., Chopp L.B., Lu Q., Shang J., Pelletier O.B., He Y. (2018). REV-ERBα Regulates TH17 Cell Development and Autoimmunity. Cell Rep..

[48] Michael A.K., Fribourgh J.L., Chelliah Y., Sandate C.R., Hura G.L., Schneidman-Duhovny D., Tripathi S.M., Takahashi J.S., Partch C.L. (2017). Formation of a Repressive Complex in the Mammalian Circadian Clock Is Mediated by the Secondary Pocket of CRY1. Proc. Natl. Acad. Sci. USA.

[49] Rosensweig C., Reynolds K.A., Gao P., Laothamatas I., Shan Y., Ranganathan R., Takahashi J.S., Green C.B. (2018). An Evolutionary Hotspot Defines Functional Differences between CRYPTOCHROMES. Nat. Commun..

[50] Olkkonen J., Kouri V.-P., Kuusela E., Ainola M., Nordström D., Eklund K.K., Mandelin J. (2017). DEC2 Blocks the Effect of the ARNTL2/NPAS2 Dimer on the Expression of PER3 and DBP. J. Circadian Rhythm..

[51] Ding J., Chen P., Qi C. (2024). Circadian Rhythm Regulation in the Immune System. Immunology.

[52] Takahashi J.S. (2017). Transcriptional Architecture of the Mammalian Circadian Clock. Nat. Rev. Genet..

[53] Sato T.K., Panda S., Miraglia L.J., Reyes T.M., Rudic R.D., McNamara P., Naik K.A., FitzGerald G.A., Kay S.A., Hogenesch J.B. (2004). A Functional Genomics Strategy Reveals Rora as a Component of the Mammalian Circadian Clock. Neuron.

[54] Zhang Y., Fang B., Emmett M.J., Damle M., Sun Z., Feng D., Armour S.M., Remsberg J.R., Jager J., Soccio R.E. (2015). GENE REGULATION. Discrete Functions of Nuclear Receptor Rev-Erbα Couple Metabolism to the Clock. Science.

[55] Sciarra F., Franceschini E., Campolo F., Gianfrilli D., Pallotti F., Paoli D., Isidori A.M., Venneri M.A. (2020). Disruption of Circadian Rhythms: A Crucial Factor in the Etiology of Infertility. Int. J. Mol. Sci..

[56] Petrenko V., Sinturel F., Riezman H., Dibner C. (2023). Lipid Metabolism around the Body Clocks. Prog. Lipid Res..

[57] Ripperger J.A., Schibler U. (2006). Rhythmic CLOCK-BMAL1 Binding to Multiple E-Box Motifs Drives Circadian Dbp Transcription and Chromatin Transitions. Nat. Genet..

[58] Stratmann M., Stadler F., Tamanini F., van der Horst G.T.J., Ripperger J.A. (2010). Flexible Phase Adjustment of Circadian Albumin D Site-Binding Protein (DBP) Gene Expression by CRYPTOCHROME1. Genes Dev..

[59] Ueda H.R., Hayashi S., Chen W., Sano M., Machida M., Shigeyoshi Y., Iino M., Hashimoto S. (2005). System-Level Identification of Transcriptional Circuits Underlying Mammalian Circadian Clocks. Nat. Genet..

[60] Sartor F., Ferrero-Bordera B., Haspel J., Sperandio M., Holloway P.M., Merrow M. (2024). Circadian Clock and Hypoxia. Circ. Res..

[61] Pati P., Valcin J.A., Zhang D., Neder T.H., Millender-Swain T., Allan J.M., Sedaka R., Jin C., Becker B.K., Pollock D.M. (2021). Liver Circadian Clock Disruption Alters Perivascular Adipose Tissue Gene Expression and Aortic Function in Mice. Am. J. Physiol.-Regul. Integr. Comp. Physiol..

[62] Frazier K., Manzoor S., Carroll K., DeLeon O., Miyoshi S., Miyoshi J., St George M., Tan A., Chrisler E.A., Izumo M. (2023). Gut Microbes and the Liver Circadian Clock Partition Glucose and Lipid Metabolism. J. Clin. Investig..

[63] Zhang D., Tong X., Nelson B.B., Jin E., Sit J., Charney N., Yang M., Omary M.B., Yin L. (2018). The Hepatic BMAL1/AKT/Lipogenesis Axis Protects Against Alcoholic Liver Disease in Mice via Promoting PPARα Pathway. Hepatology.

[64] Canaple L., Rambaud J., Dkhissi-Benyahya O., Rayet B., Tan N.S., Michalik L., Delaunay F., Wahli W., Laudet V. (2006). Reciprocal Regulation of Brain and Muscle Arnt-like Protein 1 and Peroxisome Proliferator-Activated Receptor Alpha Defines a Novel Positive Feedback Loop in the Rodent Liver Circadian Clock. Mol. Endocrinol..

[65] Duszka K., Wahli W. (2020). Peroxisome Proliferator-Activated Receptors as Molecular Links between Caloric Restriction and Circadian Rhythm. Nutrients.

[66] Li M.-D., Li C.-M., Wang Z. (2012). The Role of Circadian Clocks in Metabolic Disease. Yale J. Biol. Med..

[67] Yang Y., Yang T., Zhao Z., Zhang H., Yuan P., Wang G., Zhao Z., An J., Lyu Z., Xing J. (2022). Down-Regulation of BMAL1 by MiR-494-3p Promotes Hepatocellular Carcinoma Growth and Metastasis by Increasing GPAM-Mediated Lipid Biosynthesis. Int. J. Biol. Sci..

[68] Pan X., Bradfield C.A., Hussain M.M. (2016). Global and Hepatocyte-Specific Ablation of Bmal1 Induces Hyperlipidaemia and Enhances Atherosclerosis. Nat. Commun..

[69] Ma D., Liu T., Chang L., Rui C., Xiao Y., Li S., Hogenesch J.B., Chen Y.E., Lin J.D. (2015). The Liver Clock Controls Cholesterol Homeostasis through Trib1 Protein-Mediated Regulation of PCSK9/Low Density Lipoprotein Receptor (LDLR) Axis. J. Biol. Chem..

[70] Ramos C.A., Ouyang C., Qi Y., Chung Y., Cheng C.-T., LaBarge M.A., Seewaldt V.L., Ann D.K. (2020). A Non-Canonical Function of BMAL1 Metabolically Limits Obesity-Promoted Triple-Negative Breast Cancer. iScience.

[71] Pan X., Jiang X.-C., Hussain M.M. (2013). Impaired Cholesterol Metabolism and Enhanced Atherosclerosis in Clock Mutant Mice. Circulation.

[72] Turek F.W., Joshu C., Kohsaka A., Lin E., Ivanova G., McDearmon E., Laposky A., Losee-Olson S., Easton A., Jensen D.R. (2005). Obesity and Metabolic Syndrome in Circadian Clock Mutant Mice. Science.

[73] Pan X., Queiroz J., Hussain M.M. (2020). Nonalcoholic Fatty Liver Disease in CLOCK Mutant Mice. J. Clin. Investig..

[74] Ribas-Latre A., Santos R.B., Fekry B., Tamim Y.M., Shivshankar S., Mohamed A.M.T., Baumgartner C., Kwok C., Gebhardt C., Rivera A. (2021). Cellular and Physiological Circadian Mechanisms Drive Diurnal Cell Proliferation and Expansion of White Adipose Tissue. Nat. Commun..

[75] Rudic R.D., McNamara P., Curtis A.-M., Boston R.C., Panda S., Hogenesch J.B., FitzGerald G.A. (2004). BMAL1 and CLOCK, Two Essential Components of the Circadian Clock, Are Involved in Glucose Homeostasis. PLoS Biol..

[76] Pan X., Hussain M.M. (2009). Clock Is Important for Food and Circadian Regulation of Macronutrient Absorption in Mice. J. Lipid Res..

[77] Pan X., Zhang Y., Wang L., Hussain M.M. (2010). Diurnal Regulation of MTP and Plasma Triglyceride by CLOCK Is Mediated by SHP. Cell Metab..

[78] Solt L.A., Wang Y., Banerjee S., Hughes T., Kojetin D.J., Lundasen T., Shin Y., Liu J., Cameron M.D., Noel R. (2012). Regulation of Circadian Behaviour and Metabolism by Synthetic REV-ERB Agonists. Nature.

[79] Le Martelot G., Claudel T., Gatfield D., Schaad O., Kornmann B., Lo Sasso G., Moschetta A., Schibler U. (2009). REV-ERBalpha Participates in Circadian SREBP Signaling and Bile Acid Homeostasis. PLoS Biol..

[80] Brown S.A. (2016). Circadian Metabolism: From Mechanisms to Metabolomics and Medicine. Trends Endocrinol. Metab..

[81] Fang B., Lazar M.A. (2015). Dissecting the Rev-Erbα Cistrome and the Mechanisms Controlling Circadian Transcription in Liver. Cold Spring Harb. Symp. Quant. Biol..

[82] Feng D., Liu T., Sun Z., Bugge A., Mullican S.E., Alenghat T., Liu X.S., Lazar M.A. (2011). A Circadian Rhythm Orchestrated by Histone Deacetylase 3 Controls Hepatic Lipid Metabolism. Science.

[83] Gréchez-Cassiau A., Feillet C., Guérin S., Delaunay F. (2015). The Hepatic Circadian Clock Regulates the Choline Kinase α Gene through the BMAL1-REV-ERBα Axis. Chronobiol. Int..

[84] Acosta-Rodríguez V.A., Márquez S., Salvador G.A., Pasquaré S.J., Gorné L.D., Garbarino-Pico E., Giusto N.M., Guido M.E. (2013). Daily Rhythms of Glycerophospholipid Synthesis in Fibroblast Cultures Involve Differential Enzyme Contributions. J. Lipid Res..

[85] Pan X., Mota S., Zhang B. (2020). Circadian-Clock Regulation on Lipid Metabolism and Metabolic Diseases. Adv. Exp. Med. Biol..

[86] Wang T., Yang P., Zhan Y., Xia L., Hua Z., Zhang J. (2013). Deletion of Circadian Gene Per1 Alleviates Acute Ethanol-Induced Hepatotoxicity in Mice. Toxicology.

[87] Grimaldi B., Bellet M.M., Katada S., Astarita G., Hirayama J., Amin R.H., Granneman J.G., Piomelli D., Leff T., Sassone-Corsi P. (2010). PER2 Controls Lipid Metabolism by Direct Regulation of PPARγ. Cell Metab..

[88] Sato F., Kohsaka A., Bhawal U.K., Muragaki Y. (2018). Potential Roles of Dec and Bmal1 Genes in Interconnecting Circadian Clock and Energy Metabolism. Int. J. Mol. Sci..

[89] Shen L., Cui A., Xue Y., Cui Y., Dong X., Gao Y., Yang H., Fang F., Chang Y. (2014). Hepatic Differentiated Embryo-Chondrocyte-Expressed Gene 1 (Dec1) Inhibits Sterol Regulatory Element-Binding Protein-1c (Srebp-1c) Expression and Alleviates Fatty Liver Phenotype. J. Biol. Chem..

[90] Liu C., Li S., Liu T., Borjigin J., Lin J.D. (2007). Transcriptional Coactivator PGC-1alpha Integrates the Mammalian Clock and Energy Metabolism. Nature.

[91] Yang X., Chen X., Wang W., Qu S., Lai B., Zhang J., Chen J., Han C., Tian Y., Xiao Y. (2024). Transcriptional Profile of Human Thymus Reveals IGFBP5 Is Correlated with Age-Related Thymic Involution. Front. Immunol..

[92] Yang Y., Wei H., Song T., Cai A., Zhou Y., Peng J., Jiang S., Peng J. (2017). E4BP4 Mediates Glucocorticoid-Regulated Adipogenesis through COX2. Mol. Cell. Endocrinol..

[93] O’Keeffe M., St-Onge M.-P. (2012). Sleep Duration and Disorders in Pregnancy: Implications for Glucose Metabolism and Pregnancy Outcomes. Int. J. Obes..

[94] Kettner N.M., Voicu H., Finegold M.J., Coarfa C., Sreekumar A., Putluri N., Katchy C.A., Lee C., Moore D.D., Fu L. (2016). Circadian Homeostasis of Liver Metabolism Suppresses Hepatocarcinogenesis. Cancer Cell.

[95] Lamberg L. (2006). Sleeping Poorly While Pregnant May Not Be “Normal”. JAMA.

[96] Fu Q. (2018). Hemodynamic and Electrocardiographic Aspects of Uncomplicated Singleton Pregnancy. Adv. Exp. Med. Biol..

[97] Okun M.L., Schetter C.D., Glynn L.M. (2011). Poor Sleep Quality Is Associated with Preterm Birth. Sleep.

[98] Lee K.A., Zaffke M.E., McEnany G. (2000). Parity and Sleep Patterns during and after Pregnancy. Obstet. Gynecol..

[99] Zhao P., Bedrick B.S., Brown K.E., McCarthy R., Chubiz J.E., Ju Y.-E.S., Raghuraman N., Fay J.C., Jungheim E.S., Herzog E.D. (2022). Sleep Behavior and Chronotype before and throughout Pregnancy. Sleep Med..

[100] Sedov I.D., Cameron E.E., Madigan S., Tomfohr-Madsen L.M. (2018). Sleep Quality during Pregnancy: A Meta-Analysis. Sleep Med. Rev..

[101] Facco F.L., Kramer J., Ho K.H., Zee P.C., Grobman W.A. (2010). Sleep Disturbances in Pregnancy. Obstet. Gynecol..

[102] Rawal S., Hinkle S.N., Zhu Y., Albert P.S., Zhang C. (2017). A Longitudinal Study of Sleep Duration in Pregnancy and Subsequent Risk of Gestational Diabetes: Findings from a Prospective, Multiracial Cohort. Am. J. Obstet. Gynecol..

[103] Mindell J.A., Cook R.A., Nikolovski J. (2015). Sleep Patterns and Sleep Disturbances across Pregnancy. Sleep Med..

[104] Hillier S.E., Olander E.K. (2017). Women’s Dietary Changes before and during Pregnancy: A Systematic Review. Midwifery.

[105] Clarke G.S., Gatford K.L., Young R.L., Grattan D.R., Ladyman S.R., Page A.J. (2021). Maternal Adaptations to Food Intake across Pregnancy: Central and Peripheral Mechanisms. Obesity.

[106] Facco F.L., Grobman W.A., Reid K.J., Parker C.B., Hunter S.M., Silver R.M., Basner R.C., Saade G.R., Pien G.W., Manchanda S. (2017). Objectively Measured Short Sleep Duration and Later Sleep Midpoint in Pregnancy Are Associated with a Higher Risk of Gestational Diabetes. Am. J. Obstet. Gynecol..

[107] Facco F.L., Parker C.B., Hunter S., Reid K.J., Zee P.C., Silver R.M., Haas D.M., Chung J.H., Pien G.W., Nhan-Chang C.-L. (2018). Association of Adverse Pregnancy Outcomes with Self-Reported Measures of Sleep Duration and Timing in Women Who Are Nulliparous. J. Clin. Sleep Med..

[108] Redfern K.M., Hine R.S., Hollands H.J., Welch C.R., Pinkney J.H., Rees G.A. (2019). Objectively Measured Sleep Duration and Plasma Glucose Values Following an Oral Glucose Tolerance Test amongst Pregnant Women with Obesity in the UK. Sleep Med..

[109] Herring S.J., Nelson D.B., Pien G.W., Homko C., Goetzl L.M., Davey A., Foster G.D. (2014). Objectively Measured Sleep Duration and Hyperglycemia in Pregnancy. Sleep Med..

[110] Wang L., Jin F. (2020). Association between Maternal Sleep Duration and Quality, and the Risk of Preterm Birth: A Systematic Review and Meta-Analysis of Observational Studies. BMC Pregnancy Childbirth.

[111] Qiu C., Sanchez S.E., Gelaye B., Enquobahrie D.A., Ananth C.V., Williams M.A. (2015). Maternal Sleep Duration and Complaints of Vital Exhaustion during Pregnancy Is Associated with Placental Abruption. J. Matern. Fetal Neonatal Med..

[112] Wang Q., Liu C., Zhang Z. (2016). Transthyretin and Normal Human Pregnancy: Mini Review. Crit. Rev. Eukaryot. Gene Expr..

[113] Herrera E. (2000). Metabolic Adaptations in Pregnancy and Their Implications for the Availability of Substrates to the Fetus. Eur. J. Clin. Nutr..

[114] Rebuffé-Scrive M., Enk L., Crona N., Lönnroth P., Abrahamsson L., Smith U., Björntorp P. (1985). Fat Cell Metabolism in Different Regions in Women. Effect of Menstrual Cycle, Pregnancy, and Lactation. J. Clin. Investig..

[115] Alvarez J.J., Montelongo A., Iglesias A., Lasunción M.A., Herrera E. (1996). Longitudinal Study on Lipoprotein Profile, High Density Lipoprotein Subclass, and Postheparin Lipases during Gestation in Women. J. Lipid Res..

[116] Zhang Z., Zhou Z., Li H. (2023). The Role of Lipid Dysregulation in Gestational Diabetes Mellitus: Early Prediction and Postpartum Prognosis. J. Diabetes Investig..

[117] Wu L., Wu Q., Li Q., Cao S., Zhang Y., Liu Y., Qin X. (2022). Consecutive Reference Intervals for Biochemical Indices Related to Serum Lipid Levels and Renal Function during Normal Pregnancy. BMC Pregnancy Childbirth.

[118] Roeters van Lennep J.E., Tokgözoğlu L.S., Badimon L., Dumanski S.M., Gulati M., Hess C.N., Holven K.B., Kavousi M., Kayıkçıoğlu M., Lutgens E. (2023). Women, Lipids, and Atherosclerotic Cardiovascular Disease: A Call to Action from the European Atherosclerosis Society. Eur. Heart J..

[119] Mead J.R., Irvine S.A., Ramji D.P. (2002). Lipoprotein Lipase: Structure, Function, Regulation, and Role in Disease. J. Mol. Med..

[120] Preiss-Landl K., Zimmermann R., Hämmerle G., Zechner R. (2002). Lipoprotein Lipase: The Regulation of Tissue Specific Expression and Its Role in Lipid and Energy Metabolism. Curr. Opin. Lipidol..

[121] Sweeting A., Wong J., Murphy H.R., Ross G.P. (2022). A Clinical Update on Gestational Diabetes Mellitus. Endocr. Rev..

[122] Barbour L.A., McCurdy C.E., Hernandez T.L., Kirwan J.P., Catalano P.M., Friedman J.E. (2007). Cellular Mechanisms for Insulin Resistance in Normal Pregnancy and Gestational Diabetes. Diabetes Care.

[123] Pujol E., Proenza A., Lladó I., Roca P. (2005). Pregnancy Effects on Rat Adipose Tissue Lipolytic Capacity Are Dependent on Anatomical Location. Cell. Physiol. Biochem..

[124] Zhang L., Zhao J., Lam S.M., Chen L., Gao Y., Wang W., Xu Y., Tan T., Yu H., Zhang M. (2024). Low-Input Lipidomics Reveals Lipid Metabolism Remodelling during Early Mammalian Embryo Development. Nat. Cell Biol..

[125] Amundsen A.L., Khoury J., Iversen P.O., Bergei C., Ose L., Tonstad S., Retterstøl K. (2006). Marked Changes in Plasma Lipids and Lipoproteins during Pregnancy in Women with Familial Hypercholesterolemia. Atherosclerosis.

[126] Bever A.M., Mumford S.L., Schisterman E.F., Sjaarda L., Perkins N.J., Gerlanc N., DeVilbiss E.A., Silver R.M., Kim K., Nobles C.J. (2020). Maternal Preconception Lipid Profile and Gestational Lipid Changes in Relation to Birthweight Outcomes. Sci. Rep..

[127] Baas R.E., Hutten B.A., Henrichs J., Vrijkotte T.G.M. (2022). Associations Between Maternal Lipid Blood Levels at the 13th Week of Pregnancy and Offspring’s Adiposity at Age 11-12 Years. J. Clin. Endocrinol. Metab..

[128] Waage C.W., Mdala I., Stigum H., Jenum A.K., Birkeland K.I., Shakeel N., Michelsen T.M., Richardsen K.R., Sletner L. (2022). Lipid and Lipoprotein Concentrations during Pregnancy and Associations with Ethnicity. BMC Pregnancy Childbirth.

[129] Khoury J., Henriksen T., Christophersen B., Tonstad S. (2005). Effect of a Cholesterol-Lowering Diet on Maternal, Cord, and Neonatal Lipids, and Pregnancy Outcome: A Randomized Clinical Trial. Am. J. Obstet. Gynecol..

[130] Chen Q., Chen H., Xi F., Sagnelli M., Zhao B., Chen Y., Yang M., Xu D., Jiang Y., Chen G. (2020). Association between Maternal Blood Lipids Levels during Pregnancy and Risk of Small-for-Gestational-Age Infants. Sci. Rep..

[131] Jin W.-Y., Lin S.-L., Hou R.-L., Chen X.-Y., Han T., Jin Y., Tang L., Zhu Z.-W., Zhao Z.-Y. (2016). Associations between Maternal Lipid Profile and Pregnancy Complications and Perinatal Outcomes: A Population-Based Study from China. BMC Pregnancy Childbirth.

[132] Xu H., Ma Y., Zhang L., Liang Z., Chen D. (2021). Impact of Pre-Pregnancy Body Mass Index, Weight Gain and Blood Lipid Level during Pregnancy on Pregnancy Outcome in Patients with Gestational Diabetes Mellitus. Zhejiang Da Xue Xue Bao Yi Xue Ban.

[133] Adank M.C., Benschop L., Peterbroers K.R., Smak Gregoor A.M., Kors A.W., Mulder M.T., Schalekamp-Timmermans S., Roeters Van Lennep J.E., Steegers E.A.P. (2019). Is Maternal Lipid Profile in Early Pregnancy Associated with Pregnancy Complications and Blood Pressure in Pregnancy and Long Term Postpartum?. Am. J. Obstet. Gynecol..

[134] Xue R.-H., Wu D.-D., Zhou C.-L., Chen L., Li J., Li Z.-Z., Fan J.-X., Liu X.-M., Lin X.-H., Huang H.-F. (2021). Association of High Maternal Triglyceride Levels Early and Late in Pregnancy with Adverse Outcomes: A Retrospective Cohort Study. J. Clin. Lipidol..

[135] Baracchi A., Piani F., Degli Esposti D., Agnoletti D., Borghi C., D’Addato S., Bologna HDP Study Group (2024). When Pregnancy-Associated Hypertriglyceridemia Goes above and beyond the Risk of Pancreatitis. Intern. Emerg. Med..

[136] Huang J., Meng X., Li J., Gong X., Wu T., Shi H., Li X., Wang X., Yang J., Zhao Y. (2024). Serum Lipid Reference Values Recommended during a Twin Pregnancy and Evaluating Its Association with Perinatal Outcomes. BMC Pregnancy Childbirth.

[137] Liu L., Zhang X., Qin K., Xu C., Ruan F., Liu Y., Zhao H., Wang Y., Xiong Y., Zhou Q. (2024). Characteristics of Serum Lipid Metabolism among Women Complicated with Hypertensive Disorders in Pregnancy: A Retrospective Cohort Study in Mainland China. Obstet. Gynecol. Int..

[138] Dimitriadis E., Menkhorst E., Saito S., Kutteh W.H., Brosens J.J. (2020). Recurrent Pregnancy Loss. Nat. Rev. Dis. Primers.

[139] Yang H.-L., Lai Z.-Z., Shi J.-W., Zhou W.-J., Mei J., Ye J.-F., Zhang T., Wang J., Zhao J.-Y., Li D.-J. (2022). A Defective Lysophosphatidic Acid-Autophagy Axis Increases Miscarriage Risk by Restricting Decidual Macrophage Residence. Autophagy.

[140] Houser B.L., Tilburgs T., Hill J., Nicotra M.L., Strominger J.L. (2011). Two Unique Human Decidual Macrophage Populations. J. Immunol..

[141] Li N., Gao R., Chen X., Liu X., Ding Y., He J., Li F., Cao X., Yang C., Wang Y. (2022). Carnitine Palmitoyltransferase 1A Is Essential for Decidualization in Mice. Theriogenology.

[142] Kagan V.E., Mao G., Qu F., Angeli J.P.F., Doll S., Croix C.S., Dar H.H., Liu B., Tyurin V.A., Ritov V.B. (2017). Oxidized Arachidonic and Adrenic PEs Navigate Cells to Ferroptosis. Nat. Chem. Biol..

[143] Pedrera L., Espiritu R.A., Ros U., Weber J., Schmitt A., Stroh J., Hailfinger S., von Karstedt S., García-Sáez A.J. (2021). Ferroptotic Pores Induce Ca^2+^ Fluxes and ESCRT-III Activation to Modulate Cell Death Kinetics. Cell Death Differ..

[144] Pope L.E. (2023). Regulation of Ferroptosis by Lipid Metabolism. Trends Cell Biol..

[145] Sun F., Cui L., Qian J., Li M., Chen L., Chen C., Li D., Wang S., Du M. (2023). Decidual Stromal Cell Ferroptosis Associated with Abnormal Iron Metabolism Is Implicated in the Pathogenesis of Recurrent Pregnancy Loss. Int. J. Mol. Sci..

[146] Li S., Zhai J., Liu J., Hong Y., Zhao W., Zhao A., Sun K., Du Y., Chen Z.-J. (2017). BMAL1 Facilitates Trophoblast Migration and Invasion via SP1-DNMT1/DAB2IP Pathway in Recurrent Spontaneous Abortion. Oncotarget.

[147] Li R., Cheng S., Wang Z. (2015). Circadian Clock Gene Plays a Key Role on Ovarian Cycle and Spontaneous Abortion. Cell. Physiol. Biochem..

[148] Muter J., Lucas E.S., Chan Y.-W., Brighton P.J., Moore J.D., Lacey L., Quenby S., Lam E.W.-F., Brosens J.J. (2015). The Clock Protein Period 2 Synchronizes Mitotic Expansion and Decidual Transformation of Human Endometrial Stromal Cells. FASEB J..

[149] Cui L., Xu F., Xu C., Ding Y., Wang S., Du M. (2022). Circadian Gene Rev-Erbα Influenced by Sleep Conduces to Pregnancy by Promoting Endometrial Decidualization via IL-6-PR-C/EBPβ Axis. J. Biomed. Sci..

[150] Cui L., Xu F., Wang S., Jiang Z., Liu L., Ding Y., Sun X., Du M. (2021). Melatonin-MT1 Signal Is Essential for Endometrial Decidualization. Reproduction.

[151] Zhen Y., Xi Z., Hu L., Chen Y., Ge L., Wei W., Loor J.J., Yang Q., Wang M. (2022). Impacts of Circadian Gene Period2 Knockout on Intestinal Metabolism and Hepatic Antioxidant and Inflammation State in Mice. Oxid. Med. Cell. Longev..

[152] Committee on Practice Bulletins—Obstetrics (2018). ACOG Practice Bulletin No. 190: Gestational Diabetes Mellitus. Obstet. Gynecol..

[153] Juan J., Yang H. (2020). Prevalence, Prevention, and Lifestyle Intervention of Gestational Diabetes Mellitus in China. Int. J. Environ. Res. Public Health.

[154] Samimi M., Jamilian M., Asemi Z., Esmaillzadeh A. (2015). Effects of Omega-3 Fatty Acid Supplementation on Insulin Metabolism and Lipid Profiles in Gestational Diabetes: Randomized, Double-Blind, Placebo-Controlled Trial. Clin. Nutr..

[155] Liu W., Gao M., Yang S., Sun C., Bi Y., Li Y., Wang J., Yuan X. (2023). Effects of Omega-3 Supplementation on Glucose and Lipid Metabolism in Patients with Gestational Diabetes: A Meta-Analysis of Randomized Controlled Trials. J Diabetes Complications.

[156] Carpentier A., Mittelman S.D., Lamarche B., Bergman R.N., Giacca A., Lewis G.F. (1999). Acute Enhancement of Insulin Secretion by FFA in Humans Is Lost with Prolonged FFA Elevation. Am. J. Physiol..

[157] Yang Y., Peng Y., Yu B., Wang H. (2024). ABHD5-CPT1B: An Important Way of Regulating Placental Lipid Metabolism in Gestational Diabetes Mellitus. Arch. Med. Res..

[158] Xu Y.-H., Shi L., Bao Y.-P., Chen S.-J., Shi J., Zhang R.-L., Lu L. (2018). Association between Sleep Duration during Pregnancy and Gestational Diabetes Mellitus: A Meta-Analysis. Sleep Med..

[159] Zhang X., Zhang R., Cheng L., Wang Y., Ding X., Fu J., Dang J., Moore J., Li R. (2020). The Effect of Sleep Impairment on Gestational Diabetes Mellitus: A Systematic Review and Meta-Analysis of Cohort Studies. Sleep Med..

[160] Pappa K.I., Gazouli M., Anastasiou E., Iliodromiti Z., Antsaklis A., Anagnou N.P. (2013). Circadian Clock Gene Expression Is Impaired in Gestational Diabetes Mellitus. Gynecol. Endocrinol..

[161] Gooley J.J., Mohapatra L., Twan D.C.K. (2017). The Role of Sleep Duration and Sleep Disordered Breathing in Gestational Diabetes Mellitus. Neurobiol. Sleep Circadian Rhythm..

[162] Newhouse L.P., Joyner M.J., Curry T.B., Laurenti M.C., Man C.D., Cobelli C., Vella A., Limberg J.K. (2017). Three Hours of Intermittent Hypoxia Increases Circulating Glucose Levels in Healthy Adults. Physiol. Rep..

[163] Garovic V.D., White W.M., Vaughan L., Saiki M., Parashuram S., Garcia-Valencia O., Weissgerber T.L., Milic N., Weaver A., Mielke M.M. (2020). Incidence and Long-Term Outcomes of Hypertensive Disorders of Pregnancy. J. Am. Coll. Cardiol..

[164] Szilagyi J.T., Avula V., Fry R.C. (2020). Perfluoroalkyl Substances (PFAS) and Their Effects on the Placenta, Pregnancy, and Child Development: A Potential Mechanistic Role for Placental Peroxisome Proliferator-Activated Receptors (PPARs). Curr. Environ. Health Rep..

[165] Lai W., Yu L., Deng Y. (2024). PPARγ Alleviates Preeclampsia Development by Regulating Lipid Metabolism and Ferroptosis. Commun. Biol..

[166] Shin E.-K., Kang H.Y., Yang H., Jung E.-M., Jeung E.-B. (2016). The Regulation of Fatty Acid Oxidation in Human Preeclampsia. Reprod. Sci..

[167] Khaire A.A., Thakar S.R., Wagh G.N., Joshi S.R. (2021). Placental Lipid Metabolism in Preeclampsia. J. Hypertens..

[168] Stadler J.T., Scharnagl H., Wadsack C., Marsche G. (2023). Preeclampsia Affects Lipid Metabolism and HDL Function in Mothers and Their Offspring. Antioxidants.

[169] Aizawa M., Murakami K., Takahashi I., Onuma T., Noda A., Ueno F., Matsuzaki F., Ishikuro M., Obara T., Hamada H. (2022). Skipping Breakfast during Pregnancy and Hypertensive Disorders of Pregnancy in Japanese Women: The Tohoku Medical Megabank Project Birth and Three-Generation Cohort Study. Nutr. J..

[170] Li Y., Li J., Hou Y., Huang L., Bian Y., Song G., Qiao C. (2020). Circadian Clock Gene Clock Is Involved in the Pathogenesis of Preeclampsia through Hypoxia. Life Sci..

[171] Lu Q., Zhang X., Wang Y., Li J., Xu Y., Song X., Su S., Zhu X., Vitiello M.V., Shi J. (2021). Sleep Disturbances during Pregnancy and Adverse Maternal and Fetal Outcomes: A Systematic Review and Meta-Analysis. Sleep Med. Rev..

[172] Bonzini M., Palmer K.T., Coggon D., Carugno M., Cromi A., Ferrario M.M. (2011). Shift Work and Pregnancy Outcomes: A Systematic Review with Meta-Analysis of Currently Available Epidemiological Studies. BJOG.

[173] Wergeland E., Strand K. (1997). Working Conditions and Prevalence of Pre-Eclampsia, Norway 1989. Int. J. Gynaecol. Obstet..

